# Recommendations for standard criteria for the positional and morphological evaluation of temporomandibular joint osseous structures using cone-beam CT: a systematic review

**DOI:** 10.1007/s00330-023-10248-4

**Published:** 2023-10-25

**Authors:** Abeer A. Almashraqi, Boshra A. Sayed, Lujain K. Mokli, Sarah A. Jaafari, Esam Halboub, Sameena Parveen, Mohammed Sultan Al-Ak’hali, Maged S. Alhammadi

**Affiliations:** 1 Department of Pre-Clinical Oral Health Sciences, College of Dental Medicine, QU Health, Qatar University, Doha, Qatar; 2 Primary Health Care, Ministry of Health, Jazan, Saudi Arabia; 3 Saudi Board of Periodontics, King Saud University, Riyadh, Saudi Arabia; 4 Saudi Board of Orthodontics and Dentofacial Orthopedics, Vision Colleges, Jeddah, Saudi Arabia; 5 Department of Maxillofacial Surgery and Diagnostic Sciences, College of Dentistry, Jazan University, Jazan, Saudi Arabia; 6 Department of Preventive Dental Sciences, College of Dentistry, Jazan University, Jazan, Saudi Arabia

**Keywords:** Cone-beam computed tomography, Comprehensiveness, Reliability, Systematic review, Temporomandibular joint osseous component

## Abstract

**Objective:**

This systematic review aimed to appraise the reliability and comprehensiveness of imaging methods in studies that used three-dimensional assessment of the temporomandibular joint (TMJ) in order to propose a standardized imaging method.

**Methods:**

Six databases/search engines were searched up until September 2022. The outcomes of interest included measurements of the mandibular condyle, glenoid fossa, joint spaces, or the entire TMJ. Two checklists were utilized: one to assess the risk of bias, with a maximum score of 37, and the other, a pre-designed checklist consisting of 22 items to evaluate the comprehensiveness of the methods used, with a maximum score of 33.

**Results:**

Out of the 2567 records retrieved, only 14 studies, which used cone bean computed tomography (CBCT), were deemed eligible and thus included in the qualitative analysis. Three studies were deemed of low risk of bias, while the remaining studies were rated as moderate to high risk of bias, primarily due to improper reporting of inter-observer agreement, varying reliability values, and a limited number of cases included in the reliability analysis. Regarding the comprehensiveness of the methods used, only four studies achieved relatively high scores. The deficiencies observed were related to the reporting of variables such as slice thickness and voxel size, absence of or improper reporting of intra- and inter-examiner reliability analyses, and failure to assess all osseous components of the TMJ.

**Conclusion:**

CBCT-based methods used to assess the positions and morphology of TMJ bony structures appear to be imperfect and lacking in comprehensiveness. Hence, criteria for a standardized assessment method of these TMJ structures are proposed.

**Clinical relevance statement:**

Accurately, comprehensively, and reliably assessing the osseous structures of the temporomandibular joint will provide valid and valuable diagnostic features of the normal temporomandibular joint, and help establish potential associations between these osseous features and temporomandibular disorders.

**Registration:**

The protocol for this systematic review was registered at the International Prospective Register of Systematic Reviews (PROSPERO, No.: CRD42020199792).

**Key Points:**

•*Although many methods have been introduced to assess the osseous structure of the temporomandibular joint, they yielded inconsistent findings*.

•*None of the published studies comprehensively assessed the temporomandibular joint*.

•*Recommendations for a comprehensive temporomandibular joint osseous assessment method were suggested for better validity and reliability of future research*.

**Supplementary Information:**

The online version contains supplementary material available at 10.1007/s00330-023-10248-4.

## Introduction

### Rationale

The temporomandibular joint (TMJ) is a unique structure as it is the only diarthrodial synovial joint in the body. Three-dimensional (3D) technology provides improved visualization of the TMJ structures. Magnetic resonance imaging (MRI) is primarily used for evaluating the TMJ’s soft tissue [1], whereas computed tomography (CT) and cone-beam computed tomography (CBCT) are primarily employed for assessing the hard tissue. Indeed, these two technologies enable a more precise analysis of the TMJ’s osseous components than ever before [1–4]. It is worth noting, however, that CBCT exposes patients to less radiation compared to CT. Studies have reported that high-resolution images can achieve excellent accuracy in TMJ examinations [5–7].

Recently, the American Academy of Oral and Maxillofacial Radiology and the American Academy of Orofacial Pain issued recommendations regarding TMJ imaging. According to these recommendations, “maxillofacial CBCT allows for evaluating the osseous and dental hard tissue components. However, due to its limited ability to distinguish soft tissue details, CBCT is inadequate for assessing the necessary information required for diagnosing and managing patients with temporomandibular disorders (TMDs)” [8].

A wide range of methods has been proposed to evaluate various aspects of the TMJ, including the dimensions, inclinations, positions, surface areas, volume of the mandibular condyles, as well as the dimensions, inclinations, and dimensional/volumetric parameters of the glenoid fossa and TMJ spaces, respectively. However, the utilization of these methods has complicated TMJ analyses. These techniques have been employed in the context of patients with TMDs, normal TMJ patients, and even to assess the effects of specific interventions on TMJ morphology and dimensions. Unfortunately, the findings derived from these studies have been inconsistent and even contradictory [9–14]. These discrepancies can be attributed to a lack of methodological consensus. For instance, while the imaging is 3D, the TMJ measurements are often taken from slice sections resulting in two-dimensional (2D) data. Furthermore, there is considerable variability in the TMJ measurements utilized for assessing the osseous structures, and there is a wide diversity in the selected study samples.

To date, there has been neither comprehensive systematic review assessing the reliability and comprehensiveness of CT or CBCT-based osseous measurements of the TMJ, nor an examination of the extent to which the existing methodologies are valid for evaluating this intricate structure.

### Objectives

This systematic review aimed to appraise the reliability and comprehensiveness of CT or CBCT-based methods employed for 3D positional and morphological assessment of the TMJ. Additionally, the review aimed to propose a standardized method for such assessments.

## Methods

### Protocol registration

The study protocol was registered with PROSPERO under registration number CRD42020199792 and was conducted following the guidelines outlined in the Cochrane Oral Health Group’s Handbook for Systematic Reviews (http://ohg.cochrane.org).

### PICOS question and eligibility criteria

The inclusion criteria for this study comprised observational studies, primarily descriptive (either retrospective or prospective), that utilized CT or CBCT for imaging purposes in adult human subjects. These studies evaluated the reliability and comprehensiveness of osseous measurements in the TMJ. On the other hand, interventional studies, case reports, case series, literature reviews, systematic reviews, opinion articles, book chapters, as well as studies involving populations other than normal adult humans (such as cadavers, animals, growing patients, individuals with craniofacial anomalies, the TMDs, trauma to the temporal or temporomandibular region, or a history of surgical intervention/s in the TMJ or surrounding area) were excluded. Additionally, records focusing on other imaging methods or outcomes were also excluded.

### Information sources, search strategy, and study selection

In August 2020, three co-authors (BS, LM, and SJ) conducted an independent and thorough search across six search engines/databases, namely PubMed, Scopus, Science Direct, Web of Science, Cochrane, and LILACS. The search was further complemented by a manual examination of the reference lists of the included studies. The same three co-authors subsequently updated the search in September 2022.

The retrieved records were entered into an Excel sheet (version 2010) to identify and remove duplicates. Subsequently, the titles and abstracts of the remaining records were screened to determine their potential for inclusion, and irrelevant studies were excluded. The full texts of the remaining studies were thoroughly reviewed, and any studies deemed irrelevant were eliminated. Two co-authors (AA and SP) carried out these steps independently. All co-authors independently assessed the potentially included studies to ensure they met the predefined inclusion criteria. In the event of disagreements, a discussion was initiated to reach a final consensus. The reporting of this systematic review adheres to the guidelines outlined in the Preferred Reporting Items for Systematic Reviews and Meta-Analyses (PRISMA) statement [15].

### Data collection

Using a pre-designed template, three co-authors (BS, LM, and SJ) independently extracted the necessary data. In cases where doubts or uncertainties arose, a fourth co-author(MA) was consulted for resolution. The extracted data encompassed two primary categories: CBCT parameters and the demographic, qualitative, and quantitative characteristics of the included studies. The findings are organized and presented in Tables 1 and 2.

### Outcome assessment

The parameter outcomes encompassed measurements of the three primary osseous components: the mandibular condyle, glenoid fossa, and TMJ spaces. Supplementary Material I provides detailed definitions of these parameters.

### Risk of bias/quality assessment of the included studies

The assessment of the risk of bias was conducted independently by three co-authors (EH, AA, and MA) using a modified checklist derived from previous studies [16–18]. Any disagreements that arose during the assessment were resolved through discussion among the co-authors. The checklist consisted of 18 items that examined the methodological robustness of the study sample and the data analysis. The maximum achievable score on the checklist was 37. Based on their scores, the studies were categorized as having a high, medium, or low risk of bias if they fell below 18, between 19 and 27, or between 28 and 37, respectively. Further details can be found in Table 3.

### The comprehensiveness of the methods used in the included studies

The term “comprehensiveness” refers to several aspects, including the view utilized for TMJ measurements (multiplanar or volumetric), the assessed TMJ components, and the variation in measurement types (linear, angular, surface area, or volumetric variables). To evaluate the comprehensiveness of the measurement methods employed for TMJ osseous components, a checklist comprising 22 items was developed. The maximum achievable score on this checklist was 33. One item focused on the reference plane or line used (maximum score of 4). Eight items addressed condylar measurements (maximum score of 12), while other eight items covered glenoid fossa measurements (maximum score of 12). The remaining five items pertained to joint space measurements (maximum score of 5) (Table 4).

### Analyses

The data were subjected to qualitative analysis. Due to significant inconsistencies in reporting the outcomes of interest across the included studies, quantitative analysis (meta-analysis) was not feasible.

## Results

### Study selection

**Fig. 1:**
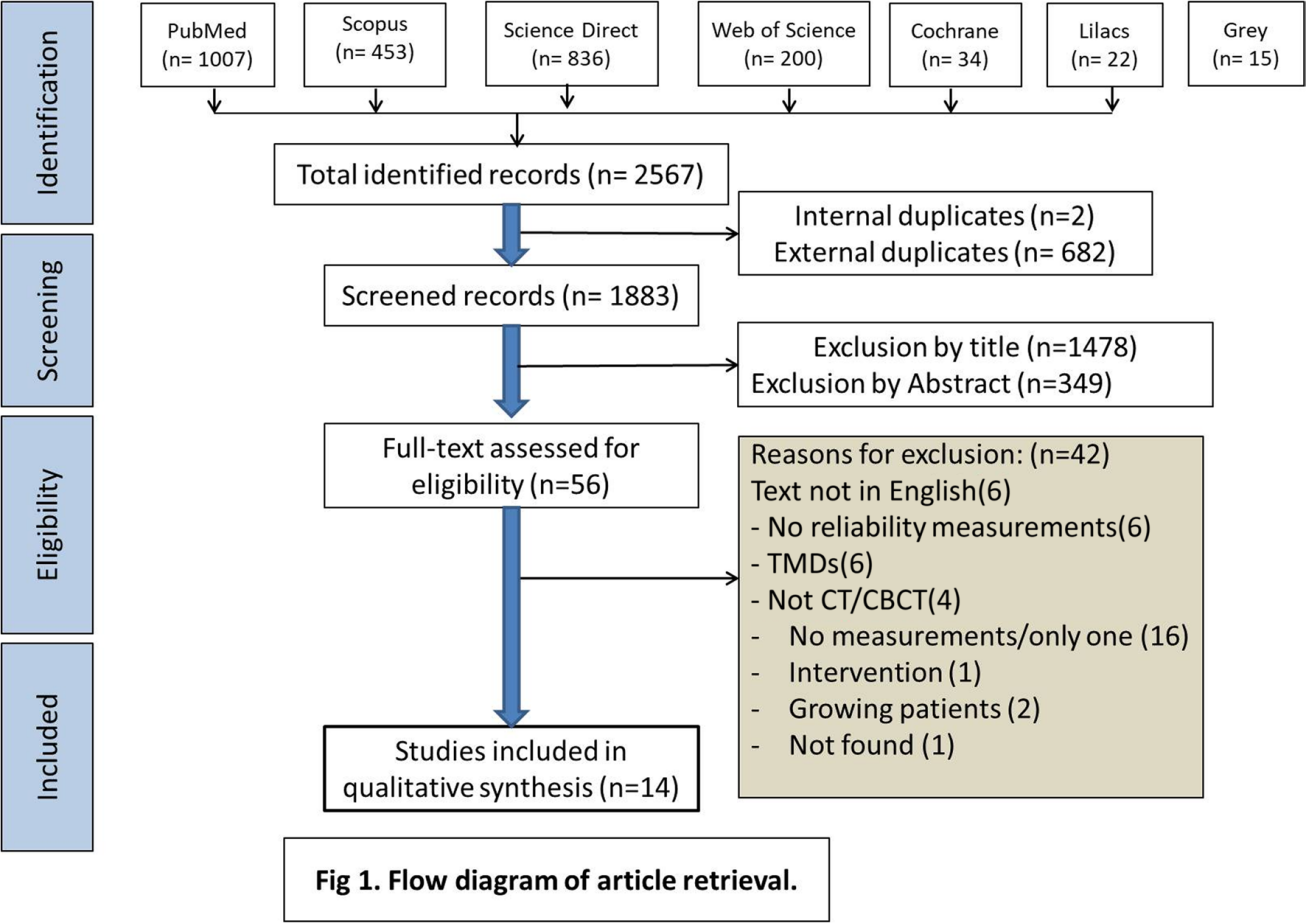
PRISMA diagram of article retrieval

The PRISMA flowchart (Fig. 1) illustrates the resulting process. Initially, 2567 records were retrieved, out of which 684 were duplicates and subsequently excluded. After screening the remaining 1883 records based on titles and abstracts, 1827 were deemed irrelevant to the review question and excluded. The full texts of the remaining 56 studies were meticulously read, leading to the exclusion of 42 studies. Ultimately, a total of 14 studies were included in the qualitative synthesis. Notably, none of these studies utilized CT.

### Study characteristics

Tables 1 and 2 provide details regarding the characteristics of the 14 studies included in the analysis. These studies were published between 2011 and 2022. Notably, no studies utilizing CT were identified. Instead, all included studies employed CBCT as the imaging modality, utilizing various machines and equipment. The Next-generation i-CAT CBCT and KaVo 3DeXam CT systems were the most frequently reported machines. The CBCT settings are outlined in Table 1. It is worth noting that there was substantial diversity in the reporting of these settings across the studies. While three studies [19–21] did not provide basic details, the remaining studies present most of the setting parameters, which are also included in Table 1.

**Table 1:** The parameters of the used CT or CBCT machines in the included studies

Author (year) [reference]	CBCT Machine	Field of View (cm)	Tube Current (mA)	Tube Potential (kVp)	Exposure Time (sec)	Resolution/Voxel size (mm)	Slice Thickness
Endo et al. 2011 [22]	Aquilion	23	100 mAs	120	NM	NM	1 mm
Alhammadi et al. 2014 [27]	Next-generation i-CAT CBCT unit	17	18.54 mAs	120	8.9	0.3	1 mm
Al-koshab et al. 2015 [25]	i-CAT Imaging System	NM	3 to 7 mA	120	20	0.3	NM
Alhammadi et al. 2016a [19]	Next-generation i-CAT CBCT unit	NM	NM	NM	NM	NM	NM
Alhammadi et al. 2016b [20]	Next-generation i-CAT CBCT unit	NM	NM	NM	NM	NM	NM
Huang et al. 2017 [29]	KaVo Dental GmbH	NM	5 mA	120	8.9	0.4	NM
Lobo et al. 2019 [32]	Next Generation i-Cat	17*23	38 mA	120	NM	0.3	NM
Chae et al. 2020 [31]	PSR 9000N TM dental CT system	FOF = 19.94; Scan size = 200*179	60 mAs	80	17	0.39	NM
García-Díaz et al. 2020 [26]	Carestream CS equipment in a 9300 computer with digital sensor	17*13.5	4 mA	90	30	0.3	NM
Serindere et al. 2020 [21]	NM	NM	NM	NM	NM	NM	NM
Ahmed et al. 2021 [24]	Planmeca ProMax 3D mid	20*17	8 mA	90	NM	NM	0.4 mm
Al-hadad et al. 2022 [28]	i-CAT Image System	16*13 cm	18.54 mAs	120	8.9	0.3	NM
Chen et al. 2022a [23]	KaVo 3DeXam CT system	16*17 cm	5 mA	120	8.9	0.4	0.5 mm
Chen et al. 2022b [30]	KaVo 3DeXam CT system	16*17 cm	5 mA	120	NM	NM	0.5 mm

*NM* Not Mentioned

**Table 2:** The demographic, qualitative and quantitative characteristics of the included studies

Author (year) [reference]	Sample size	Gender M/F	Age	Control	Measurements (linear, angular, surface area volume)	Assessed components (condyle, fossa, joint space)	Reliability test used and statistics	Imaging software
Endo et al. 2011 [22]	29	0/29	24.8 ± 5.4 and 25.1 ± 2.4	Mandibular asymmetry versus mandibular symmetry	Linear, angular, and surface area	Condyle and fossa	ICC, and paired *t*-test	volume-rendering software VG Studio MAX 1.2 and image measurement software 3D-Rugle
Alhammadi et al. 2014 [27]	90	NM	18−25	NM	Linear, angular, and surface area	Condyle, fossa and joint space	ICC	Anatomage software version 5.01
Al-koshab et al. 2015 [25]	100	50/50	30.5 (18−45)	Malay and Chinese	Linear and volume	Condyle and joint space	ICC	i-CAT classic software (linear measurements) and MIMICS 16.0 software (Condylar volume)
Alhammadi et al. 2016a [19]	60	NM	18−25	Long, average, and short faces	Linear, and angular	Condyle, fossa, joint space	ICC	Anatomage software version 5.01
Alhammadi et al. 2016b [20]	60	NM	18−25	Class I, Class II, and Class III	Linear, angular, and surface area	Condyle, fossa, and joint space	ICC	Anatomage software version 5.01
Huang et al. 2017 [29]	32	22/10	18.6	Right versus left side	Linear, angular, and volumetric	Condyle and fossa	ICC	NM
Lobo et al. 2019 [32]	180	90/90	> 18	Male versus Female, < 40 versus > 40, Class I versus class II versus class III	Linear, angular	Condyle, fossa, joint space	ICC	NM
Chae et al. 2020 [31]	120	56/64	10− < 20	Class I, Class II, Class III and normodivergent, hypodivergent, hyperdivergent	Linear and angular	Condyle, fossa, joint space	ICC	Simplant Pro 2011 pack software
García-Díaz et al. 2020 [26]	188	68/120	35.57 ± 11.61	Class I and Class II	Linear	Condyle, fossa	ICC and Kappa	3D Imaging Carestream Software CS v3.5.18 – 2015
Serindere et al. 2020 [21]	125	59/66	40.64	Male versus female, different age groups	Linear, angular, surface area, and volume	Condyle and joint space	ICC	Image Analysis Program and InVesalius software
Ahmed et al. 2021 [24]	119	60/59	18−50	Above and below 25 years old	Linear, and volume	Condyle and joint space	ICC	Romexis software version 4.6.2
Al-hadad et al. 2022 [28]	80	45/35	18−30	Normal sagittal skeletal relation	Linear, angular, and volume	Condyle, fossa, and joint space	ICC	Invivo 6/Anatomage dental software program
Chen et al. 2022a [23]	300	NM	8−30	Normal (neither open-bite nor cross-bite)	Linear and angular	Condyle, fossa, and joint space	ICC	Dolphin11.9 software
Chen et al. 2022b [30]	90	28/62	18−25	Normal overjet and overbite	Linear, and angular	Condyle, fossa, and joint space	ICC	Dolphin11.9 software

*NM* Not Mentioned

The sample sizes of the included studies varied, ranging from as low as 29 participants [22] to as high as 300 participants [23]. The age range of participants was 18 years and older, and both genders were included in most studies. Linear measurements were reported in all of the included studies, while angular measurements were reported in all studies except three [24–26]. Four studies each reported surface area [20–22, 27] and volume measurements [21, 24, 25, 28, 29]. The measurements of condylar parameters were reported in all of the included studies, while parameters related to the fossa and joint space were reported in all studies except two [21, 27] not reported fossa and two [22, 26] not reported joint space. There was variability in the software programs used, with eight different programs utilized across the 14 studies. The most commonly used software was Anatomage [19, 20, 27, 28], followed by Dolphin [23, 30]. The reliability test employed in all of the included studies was the intra-class correlation coefficient (ICC). Additionally, one study incorporated a paired *t*-test [22], and another study utilized the kappa test [26]. Further details can be found in Table 2.

### Quality assessment (risk of bias)

Table 3 presents the risk of bias for the included studies. Four studies exhibited a high risk of bias [22, 24, 26, 29], while seven showed a moderate risk [20, 21, 23, 25, 28, 31, 32]. Only three studies [19, 27, 30] were found to have an overall low risk of bias. The main shortcomings observed in most studies included the absence of blinding in measurements, inadequate documentation of the examiners’ experience or professional degree, low inter-examiner agreement, limited inclusion of cases in the reliability analysis, and inadequate presentation of the reliability analyses.

**Table 3:** Quality assessment tool of the included studies

Author (year) [reference]	Study Design			Methodological soundness						
	Sample characteristic (2)	Sample size calculation (2)	Sample subject variability (1)	Head * orientation (1)	TMJ * Orientation (1)	Measurement variability * (3)	Assessed TMJ component * (2)	Random error (1)	Blind measurement (1)	Measurements interval (1)
Endo et al. 2011 [22]	1	0	1	1	0	2	2	1	0	0
Alhammadi et al. 2014 [27]	1	2	1	1	1	2	2	1	0	1
Al-koshab et al. 2015 [25]	1	0	1	1	0	2	2	1	0	0
Alhammadi et al. 2016a [19]	1	2	1	1	1	2	2	1	0	1
Alhammadi et al. 2016b [20]	1	2	1	1	1	2	2	1	0	1
Huang et al. 2017 [29]	1	0	0	1	0	3	2	1	0	1
Lobo et al. 2019 [32]	1	0	1	1	1	2	2	1	0	1
Chae et al. 2020 [31]	1	1	1	1	0	2	2	1	1	1
García-Díaz et al. 2020 [26]	2	2	0	1	0	1	1	1	0	1
Serindere et al. 2020 [21]	1	0	1	0	0	2	2	1	0	1
Ahmed et al. 2021 [24]	1	0	1	0	0	2	2	1	0	1
Al-hadad et al. 2022 [28]	1	1	1	0	0	2	2	1	0	1
Chen et al. 2022a [23]	2	1	1	1	0	3	2	1	0	1
Chen et al. 2022b [30]	2	1	1	1	0	2	2	1	0	1
Author (year) [reference]	Methodological soundness			Data analysis						Total Score (37)
	Number of examiners (2)	Examiners’ occupation * (3)		level of intra-observer agreement (3)	level of inter-observer agreement (3)	Percentage of cases included in the reliability analysis * (4)	Presentation of the reliability of variables * (5)	Presentation of the variation values of the reliability * (1)	Statistical analysis (1)	
Endo et al. 2011 [22]	0	0		3	0	2	2	0	1	**16**
Alhammadi et al. 2014 [27]	1	0		3	3	4	5	0	1	**29**
Al-koshab et al. 2015 [25]	0	0		3	0	1	5	1	1	**19**
Alhammadi et al. 2016a [19]	1	0		3	3	4	5	0	1	**29**
Alhammadi et al. 2016b [20]	1	0		3	3	4	3	0	1	**27**
Huang et al. 2017 [29]	0	0		3	0	2	3	0	1	**18**
Lobo et al. 2019 [32]	1	0		3	3	4	2	0	1	**24**
Chae et al. 2020 [31]	0	0		3	0	2	3	0	1	**20**
García-Díaz et al. 2020 [26]	0	0		3	0	2	2	0	1	**17**
Serindere et al. 2020 [21]	1	3		3	3	2	2	0	1	**23**
Ahmed et al. 2021 [24]	0	3		3	0	1	2	0	1	**18**
Al-hadad et al. 2022 [28]	1	0		3	3	4	5	0	1	**26**
Chen et al. 2022a [23]	1	0		3	3	1	2	0	1	**23**
Chen et al. 2022b [30]	1	3		3	3	4	2	0	1	**28**

*The modified item from the original risk of bias check list

1- Sample characteristic (Satisfactory = 2, partially = 1, poorly = 0)

2- Sample size calculation (Satisfactory = 2, partially = 1, poorly = 0)

3- Sample subject variability (homogenous = 1, heterogeneous = 0)

4- Head orientation (Proper = 1, improper = 0)

5- TMJ orientation (Proper = 1, improper = 0)

6- Measurement variability: linear, angular and volume measurements (one point each; total = 3)

7- Assessed TMJ component: condyle = 1, fossa and/or joint spaces = 1 (total = 2)

8- Random error (Proper = 1, improper = 0)

9- Blind measurement (Proper = 1, improper = 0)

10- Measurements interval: adequate ≥ 2 weeks = 1, inadequate (Not mentioned) = 0

11- Number of examiners (> 2 = 2, 2 or 1 = 1 or not mentioned = 0)

12- Examiners’ occupation (Experienced = 3, GP = 2, other = 1, not mentioned = 0)

13- Level of intra-observer agreement (Excellent reliability in all measurements = 3, two third = 2, half = 1, Not mentioned = 0)

14- Level of inter-observer agreement (Excellent reliability in all measurements = 3, two third = 2, half = 1, Not mentioned = 0)

15- Percentage of cases included in the reliability analysis (All cases = 4, more than 50% = 3, 20–50% = 2, less than 20% = 1)

16- Presentation of the reliability of variables (Text, average, range only, range with the minimum and maximum variables, values for all variables are mentioned=1, 2, 3, 4, 5, respectively)

17- Presentation of the variation values of the reliability (SD and/or CI = 0, not mentioned = 1).

18- Statistical analysis: appropriate for data (ICC or CCC) = 1 or other = 0

From 0 to 18 points: high level of bias; from 19 to 27 points: medium level of bias; from 28 to 37 points: low level of bias

### Characteristics of the measurements and examiners

Eight of the included studies [19–21, 23, 27, 28, 30, 32] reported that measurements were conducted by two examiners, while five studies [22, 24, 26, 29, 31] mentioned the involvement of only one examiner. One study [25] did not provide information on this aspect. Regarding intra- or inter-examiner reliability analysis, the measurements were repeated twice in most studies, except for one study [25], where they were repeated three times. The time intervals between repeated measurements varied across studies, with a 1-week interval in two studies [22, 25], a 2-week interval in nine studies [19–21, 24, 27–30, 32], a 3-week interval in two studies [23, 31], and a 1-month interval in one study [26]. It is noteworthy that only one study [31] explicitly mentioned that examiners were blinded during the measurement process, and only three studies [21, 24, 30] reported the experience or qualifications of the examiners. Interestingly, six studies [19, 20, 27, 28, 30, 32] re-examined all cases for reliability, while five studies [21, 22, 26, 29, 31] re-examined between 25 and 50% of the cases. In contrast, only three studies [23–25] reported re-examining less than 20% of the total included cases. Additionally, only two studies [26, 32] reported pre-calibrating the examiners. For more detailed information, refer to Supplementary Material II.

Supplementary Material III provides a detailed account of the reliability analysis reporting. Seven studies [22, 24–26, 29, 31, 32] did not conduct inter-examiner reliability assessments. Meanwhile, four studies [20, 21, 23, 30] performed inter-examiner reliability assessments but did not specify the exact values; they only mentioned the overall reliability. Two studies [19, 27] presented a comprehensive analysis of inter-examiner reliability for most of the included measurements.

**Table 4:** Checklist for the validity and comprehensiveness of the methods used in assessment of the JMJ osseous components

Author (year) [reference]	Reference (3 D coordinate = 4, 2D multiplanar = 2)	Condylar Dimensions							Condylar position				Condylar inclination	
		Condylar length (1)	Condylar width (1)		Condylar height (1)		Surface area (axial, sagittal and coronal) or condylar volume (1)		Point or geometric condyle position (ML, V or AP) (3)		Condyle joint position (1)	Inter-condylar distance (1)	Condylar inclination (ML, V or AP) (3)	
Endo et al. 2011 [22]	4	1	1		1		1		3		0	0	0	
Alhammadi et al. 2014 [27]	4	1	1		1		0		3		1	1	3	
Al-koshab et al. 2015 [25]	2	1	1		1		1		0		0	0	0	
Alhammadi et al. 2016a [19]	4	1	1		1		0		3		1	1	3	
Alhammadi et al. 2016b [20]	4	1	1		1		0		3		1	1	3	
Huang et al. 2017 [29]	4	0	0		1		1		1		0	0	1	
Lobo et al. 2019 [32]	2	0	0		0		0		0		1	0	1	
Chae et al. 2020 [31]	2	1	1		0		0		0		0	0	0	
García-Díaz et al. 2020 [26]	2	1	1		1		0		0		0	0	0	
Serindere et al. 2020 [21]	2	1	1		1		1		0		0	0	0	
Ahmed et al. 2021 [24]	2	1	1		1		1		0		0	0	0	
Al-hadad et al. 2022 [28]	4	1	1		1		0		3		1	0	3	
Chen et al. 2022a [23]	2	1	1		1		0		0		1	0	1	
Chen et al. 2022b [30]	2	1	1		1		0		0		1	0	1	
	Glenoid fossa Dimensions				Glenoid fossa position	Glenoid fossa inclination			Joint space measurements					Total /33
Author (year) [reference]	Fossa height (1)	Fossa width (1)	Tubercle height (1)	Surface area (axial, sagittal or coronal) (1)	Fossa position (AP, V or ML) (3)	Fossa inclination (AP, V or ML) (3)	Anterior tubercle inclination (1)	Posterior tubercle inclination (1)	Anterior joint space (1)	Posterior joint space (1)	Superior joint space (1)	Medial joint space (1)	Volume (1)	
Endo et al. 2011 [22]	1	1	0	1	3	0	1	1	0	0	0	0	0	**19**
Alhammadi et al. 2014 [27]	1	1	1	0	3	3	1	1	1	1	1	1	0	**30**
Al-koshab et al. 2015 [25]	0	0	0	0	0	0	0	0	1	1	1	0	0	**9**
Alhammadi et al. 2016a [19]	1	1	1	0	3	3	1	1	1	1	1	1	0	**30**
Alhammadi et al. 2016b [20]	1	1	1	0	3	3	1	1	1	1	1	1	0	**30**
Huang et al. 2017 [29]	1	1	0	0	3	0	0	1	0	0	0	0	0	**14**
Lobo et al. 2019 [32]	0	0	1	0	0	0	1	0	1	1	1	0	0	**9**
Chae et al. 2020 [31]	1	0	1	0	0	0	1	0	1	1	1	1	0	**11**
García-Díaz et al. 2020 [26]	0	0	1	0	0	0	0	0	0	0	0	0	0	**6**
Serindere et al. 2020 [21]	0	0	0	0	0	0	0	0	1	1	1	0	0	**9**
Ahmed et al. 2021 [24]	0	0	0	0	0	0	0	0	1	1	1	0	0	**9**
Al-hadad et al. 2022 [28]	1	1	1	0	3	3	0	0	1	1	1	1	1	**28**
Chen et al. 2022a [23]	1	1	1	0	0	0	1	0	1	1	1	0	0	**14**
Chen et al. 2022b [30]	1	1	1	0	0	0	1	0	1	1	1	0	0	**14**

*Checklist for the validity and comprehensiveness of the methods used in assessment of the osseous JMJ components

A: items scored out of 4:

- Reference plane (3 D coordinate = 4, 2D multiplanar = 2)

B: items scored out of 3:

- Point or geometric condyle position (Not assessed = 0, One view assessment = 1, two or three views assessments = 3)

- Condyle inclination (Not assessed = 0, One view assessment = 1, two or three views assessments = 3)

- Glenoid fossa position (Not assessed = 0, One view assessment = 1, two or three views assessments = 3)

- Glenoid fossa inclination (Not assessed = 0, One view assessment = 1, two or three views assessments = 3)

C: items scored out of 1:

All other items ((Not assessed = 0, assessed = 1)

Regarding intra-examiner reliability analysis, 11 studies [20–26, 29–32] conducted it, but they did not mention the exact values; they only provided information about the overall reliability. Two studies [19, 27] furnished a detailed intra-examiner reliability analysis for most of the measurements included in the study. One study presented intra- and inter-examiners reliability analysis for landmarks coordinate system rather than the measurements [28].

### The comprehensiveness of the methods used in the included studies

Table 4 displays the comprehensiveness of the measurement methods employed to assess the osseous components of the TMJ. These methods can be categorized into four main groups: (1) View(s) and reference(s) utilized, which involved either a multiplanar view (sagittal, coronal, or axial) or a 3D view. In the 3D view, landmarks were identified in the frontal, horizontal, and midsagittal planes, with the three coordinates serving as the reference points. (2) Measurements of the mandibular condyle. (3) Measurements of the glenoid fossa. (4) Measurements of the joint spaces.

Among the included studies, eight [21, 23–26, 30–32] performed nearly all measurements using multiplanar views. On the other hand, the remaining six studies [19, 20, 22, 27–29] employed a 3D view to identifying landmarks and utilized the frontal, horizontal, and midsagittal planes as references for their measurements.

Among the 12 predetermined condylar measurements, three studies [19, 20, 27] reported 11 measurements, while one study [28] reported ten measurements. The remaining studies [21–26, 29–32] reported between two and seven measurements. Regarding the 12 predetermined glenoid fossa measurements, three studies [19, 20, 27] reported 11 measurements, one study [28] reported nine measurements, and one study [22] reported eight measurements. The remaining studies [21, 23–26, 29–32] reported between 0 and 6 measurements. Out of the five predetermined joint space measurements, only one study [28] reported all of them. Four studies [19, 20, 27, 31] reported all the measurements but excluded the total joint space volume. The remaining studies [21–26, 29, 30, 32] reported between 0 and 3 measurements.

Among the total score of 33 points on the checklist used, three studies [19, 20, 27] scored 30, while one study [28] scored 28. The remaining studies scored below 60% of the overall score.

## Discussion

There is a growing interest in measuring TMJ in various contexts: observational, to provide details on specific populations or traits; and interventional, to assess the effects of specific treatments. At the interventional level, studies have evaluated the effects of different orthodontic or orthognathic surgical interventions on the positional and morphological features of the TMJ. These interventions included the use of fixed appliances with extraction therapy [10, 33] or removable functional appliances [9, 34], distalization mechanics, maxillary arch expansion therapy [35], and orthognathic surgery [36, 37]. Furthermore, the effects of different prosthetic interventions, such as dental implants and full mouth rehabilitation [38], as well as other minor restorative procedures [39], have been studied to evaluate their impact on TMJ.

At the observational level, numerous studies have assessed differences in various anteroposterior skeletal malocclusions [20, 26, 28, 40], vertical facial patterns [19, 30, 41], and transverse discrepancies [5, 23]. Furthermore, many studies assessed the TMJ differences between patients with TMDs and those without TMDs [12, 42]. However, significant discrepancies have been observed among these studies. Some of these discrepancies can be attributed to the lack of standardization in the methodologies employed, such as the use of 2D- versus 3D-based assessment methods, despite the fact that the imaging technique of interest (CBCT) inherently provides a 3D view. Other discrepancies may be due to variations in the reliability of measurements and the parameters assessed: Did they comprehensively or partially represent the TMJ? Therefore, this systematic review aims to appraise the reliability and comprehensiveness of methods used for 3D positional and morphological assessment of the TMJ, utilizing CT and CBCT, and to recommend a standardized approach for the same purpose.

There are several important technical considerations to emphasize on CBCT imaging. Image resolution largely depends on voxel size, with smaller voxel sizes providing higher image resolution [43]. The selection of voxel size should align with the study’s specific objectives. In the studies reviewed, voxel sizes in CBCT ranged from 0.3 to 0.5 mm. Given that the TMJ is a delicate and complex region, imaging with a smaller voxel size is preferable [43, 44]. Among the included studies, five of them [25–28, 32] utilized a voxel size of 0.3 mm, while other studies either used larger voxel sizes or did not specify. For high-quality images of the TMJ, it is recommended to use a voxel size no larger than 0.3 mm, especially when a large field of view is employed [43].

Another important technical aspect in CBCT imaging is slice thickness, as smaller thicknesses retain more details while larger thicknesses may result in a loss of details. For imaging the TMJ, a recommended slice thickness is 1 mm or smaller [43, 44]. Surprisingly, only five of the included studies [22–24, 27, 30] mentioned the slice thickness used, and it is worth noting that only two of them [22, 27] adhered to the recommended thickness.

The TMJ is a complex structure, both mechanically and biologically, where changes occurring in one component of the TMJ can impact the others. Movement of the mandibular condyle in any direction typically results in corresponding changes in the surrounding joint spaces and, at times, bone remodeling in the mandibular fossa encompassing this synovial joint. Furthermore, movement on one side is often accompanied by either parallel or opposite movement on the other side [45]. Consequently, when radiographically assessing the TMJ, it is essential to include the three main components: the mandibular condyle, glenoid fossa, and joint spaces. For this systematic review, studies were included on the condition that they assessed at least two TMJ components. Eight of the included studies [19, 20, 23, 27, 28, 30–32] assessed all three TMJ components, while the remaining six studies [21, 22, 24, 25, 26, 29] focused on two components. Depending on the specific aim(s) of a study, certain TMJ component(s) can be selected for evaluation as the main outcome(s). However, it is recommended to evaluate all TMJ components (at least as secondary outcomes) due to the biomechanical interactions that occur among them.

The quality of the included studies was assessed using a checklist adapted from previously published research [16–18], with modifications made to make it more flexible and aligned with the objectives of the current systematic review. These modifications encompassed aspects such as the head orientation in 3D analysis, condylar orientation in sectional-based analysis, number of TMJ components included, diversity of measurement types (linear, angular, surface area, and/or volumetric measurements), experience and professional qualification of the examiners, percentage of cases included in the reliability analysis, and presentation of reliability results for the assessed variables. By applying this rigorous checklist, only three studies were identified as having a low risk of bias [19, 27, 30].

Reliability pertains to the accuracy of the obtained data and the extent to which any measuring tool controls random errors, which is crucial for ensuring the validity of the research. However, reliability alone is not sufficient [46]. Reliability analysis is necessary for any quantitative measurements where subjectivity may be a factor. It is recommended that two pre-calibrated examiners independently conduct the measurements, and both intra- and inter-examiner reliabilities should be assessed on at least 25% of the sample [47], although including the entire sample is preferable. All included studies reported intra-examiner reliability, although it was often presented as a single value or a range. In other words, only a few studies reported intra-examiner reliability for all individual variables. The same applies to inter-examiner reliability, which was evaluated in eight studies [19–21, 23, 27, 28, 30, 32], while the remaining six studies involved only one examiner [22, 24–26, 29, 31]. Only three studies [23–25] reported conducting the reliability analysis on fewer than the recommended number of cases (less than 25% of the entire sample).

To ensure a comprehensive TMJ osseous evaluation method, three main criteria must be met. First, landmark identification of this complex should be conducted in all three planes of space to enable precise and accurate measurements rather than relying solely on a multiplanar view. Second, the analysis must encompass the entire complex, including the glenoid fossa, mandibular condyle, and TMJ spaces. Third, there should be a diverse range of measurements covering dimensions, positions, and orientations in the three planes, as well as surface area and volumetric assessments. Interestingly, none of the included studies fulfilled all three of these criteria. The maximum score obtained was 30 out of 33 points, indicating that none of the methods employed in the included studies were comprehensive. Moreover, surprisingly, ten of the included studies scored less than 60% of the maximum score. Consequently, recommended criteria for the selected TMJ osseous evaluation method are listed to establish a standardized approach for future research, enhancing the validity of the findings.

## Limitations

In addition to the limited number of studies, most exhibited a moderate to high risk of bias. The significant inconsistencies among the included studies regarding study design, reporting of reliability, and the comprehensiveness of measured outcomes made it unfeasible to conduct a meta-analysis. Another limitation of the review was the inclusion of only studies published in English. One worthy-to-mention limitation of the current study is that 3 studies among the 14 studies included in this review were authored by the one of the researcher of this study, a matter that might introduce selection and presentation biases. However, we confirm that we applied strict criteria where other co-authors (not involved in these 3 studies) were involved in the selection, assessment, and extraction processes. Besides that, 3 out of 14 studies represent small fraction of the overall evidence. Thus, the fear of bias can be considered minimal.

## Conclusions

Considering the limitations of this review, the following conclusions can be drawn: (1) None of the evaluated methods provided a comprehensive assessment of the TMJ; (2) There was significant diversity in the performance and reporting of intra- and inter-examiner reliability analyses; and (3) Considerable variation was observed in CBCT imaging settings, particularly in slice thickness and voxel size.

Recommendations were proposed for a selected TMJ osseous evaluation method to improve the validity and reliability of future research. These recommendations include the following:

A. The recommended voxel size during acquisition is 0.3 when using a large field of view.
B. The slice thickness should ideally be no more than 1 mm.
C. For precise identification, TMJ measurements should be conducted in the 3D view, with landmarks identified using the slice locator view.
D. When using the TMJ view, proper slice orientation is crucial, with the long axis of the condyle in the axial view being perpendicular to the slice cuts.
E. It is preferable to include the three main components of the TMJ in the assessment: mandibular condyle, glenoid fossa, and joint spaces.
F. Measurements should encompass morphological variations, evaluating dimensions, positions, and angulations when applicable.
G. Measurements should encompass different mathematical aspects, including linear, angular, and surface area or volume measurements.

It is recommended to apply these current recommendations and standard criteria for the positional and morphological evaluation of TMJ osseous structures using CBCT in clinical settings, particularly for patients with TMDs, to establish correlations between evaluated osseous structures and the existing disease.

### Supplementary Information

Below is the link to the electronic supplementary material.

Supplementary file1 (DOCX 63 KB)

## Abbreviations

2D: Two-dimensional
3D: Three-dimensional
CBCT: Cone-beam computed tomography
CT: Computed tomography
MRI: Magnetic resonance imaging
PRISMA: Preferred Reporting Items for Systematic Reviews and Meta-Analyses
TMDs: Temporomandibular disorders
TMJ: Temporomandibular joint

## Declarations

### Guarantor

The scientific guarantor of this publication is Abeer A. Almashraqi.

### Statistics and biometry

No complex statistical methods were necessary for this paper.

### Study subjects or cohorts overlap

No

### Methodology

•Systematic review
