# Supplementary material for: Recommendations for standard criteria for the positional and morphological evaluation of temporomandibular joint osseous structures using cone-beam CT: a systematic review
Source: Eur Radiol. 2023 Oct 25;34(5):3126–40. doi: 10.1007/s00330-023-10248-4 (PMC11126469; doi:10.1007/s00330-023-10248-4)
Supplement: Supplementary file 1 — Supplementary file1 (DOCX 63 KB) [file 330_2023_10248_MOESM1_ESM.docx]

**Recommendations for standard criteria for the positional and morphological evaluation of temporomandibular joint osseous structures using cone-beam CT: A systematic review**

**ELECTRONIC SUPPLEMENTARY MATERIAL**

**Supplementary Material I:** Definitions of the parameters (outcome measures) for the mandibular condyle, the glenoid fossa and the TMJ spaces

| **Outcome** | **Definition** |
| --- | --- |
| **Reference view of the measurements** | The view on which the measurements were done which may be multiplanar view (sagittal, coronal or axial) or three-dimensional view in which the landmarks were identified in the three planes and the three coordinates were used as references. |
| **Condylar dimensions** | |
| Condylar length | The linear distance between the most lateral and medial point of the mandibular condyle |
| Condylar width | The linear distance between the most anterior and posterior point of the mandibular condyle |
| Condylar height | Perpendicular distance between most superior point of the mandibular condyle and the suggested reference plan |
| Condylar surface area | The total surface area of the condyle in the axial, sagittal or coronal planes |
| Condylar volume | The volumetric measurement of the condyle |
| **Condylar positions and inclinations** | |
| Condyle position | The position of the condyle in reference to the opposing planes (vertical, anteroposterior and transverse planes) by using either the nearest point to the opposing plane or the geometric center of the condyle |
| Condyle joint position | The position of the condyle inside the joint in reference to the dimensions of the surrounding spaces |
| Inter-condylar distance | The distances between both condyles using medial, lateral or geometric central points as a reference |
| Condylar inclination | The inclination of the condyle in the mediolateral, vertical and anteroposterior planes |
| **Glenoid fossa dimensions** | |
| Glenoid fossa height | Perpendicular distance between most superior point of the glenoid fossa and the suggested reference plan |
| Glenoid fossa width | The distance between the anterior tubercle and the posterior wall of the glenoid fossa |
| Tubercle height | The maximum dimension of the articular tubercle in the vertical dimension |
| Glenoid fossa surface area | The total surface area of the glenoid fossa in the axial, sagittal or coronal planes |
| **Glenoid fossa positions and inclinations** | |
| Glenoid fossa position | The position of the glenoid fossa in reference to the opposing planes (vertical, anteroposterior and transverse planes) relative to the central point of the glenoid fossa |
| Glenoid fossa inclination | The inclination of the glenoid fossa in the mediolateral, vertical and anteroposterior planes |
| Anterior tubercle inclination | Inclination of the anterior wall of the glenoid fossa space |
| Posterior tubercle inclination | Inclination of the posterior wall of the glenoid fossa space |
| **Joint spaces dimensions** | |
| Anterior joint space | The shortest distance between the most anterior condylar and the most posterior fossa points |
| Posterior joint space | The shortest distance between the most posterior condylar and the most anterior fossa points |
| Superior joint space | The shortest distance between the most superior condylar and the most inferior fossa points |
| Medial joint space | The shortest distance between the most medial condylar and the most lateral fossa points |
| Joint spaces volume | The total volume of the joint spaces |

**Supplementary Material II:** Characteristics of the raters and the reliability of the measurements

| **Author (year)**  **[reference]** | **Number of examiners** | **Number of times repeated** | **Time period between repeated measurements** | **Blindness of examination** | **Qualifications of examiners** | **NO. of cases included in the reliability analysis** | **Percentage of cases included in the reliability analysis** | **Calibration of the examiner** |
| --- | --- | --- | --- | --- | --- | --- | --- | --- |
| Endo et al. 2011**^22^** | 1 | 2 | 1 week | NM | NM | 10 | 34.48 | NM |
| Alhammadi et al. 2014**^27^** | 2 | 2 | 2 weeks | NM | NM | 90 | 100 | NM |
| Al-koshab et al. 2015**^25^** | NM | 3 | 1 week | NM | NM | 10 | 10 | NM |
| Alhammadi et al. 2016 a**^19^** | 2 | 2 | 2 weeks | NM | NM | 60 | 100 | NM |
| Alhammadi et al. 2016 b**^20^** | 2 | 2 | 2 weeks | NM | NM | 60 | 100 | NM |
| Huang et al. 2017**^29^** | 1 | 2 | 2 weeks | NM | NM | 16 | 50 | NM |
| Lobo et al. 2019**^32^** | 2 | 2 | 2 weeks | NM | NM | 180 | 100 | Yes |
| Chae et al. 2020**^31^** | 1 | 2 | 3 weeks | Yes | NM | 30 | 25 | NM |
| García-Díaz et al. 2020**^26^** | 1 | 2 | 1 month | NM | NM | 60 | 31.91 | Yes against two trained examiners |
| Serindere et al. 2020**^21^** | 2 | 2 | 2 weeks | NM | Dentomaxillofacial Radiology specialists | 32 | 25.6 | NM |
| Ahmed et al. 2021**^24^** | 1 | 2 | 2 weeks | NM | Dentomaxillofacial Radiology specialists | 20 | 16.80 | NM |
| Al-hadad et al. 2022**^28^** | 2 | 2 | 2 weeks | NM | NM | 80 | 100 | NM |
| Chen et al. 2022 a**^23^** | 2 | 2 | 20 days | NM | NM | 50 | 6.7 | NM |
| Chen et al. 2022 b**^30^** | 2 | 2 | 15 days | NM | Experience of evaluating CBCT images | 90 | 100 | NM |

NM = Not Mentioned

**Supplementary material III:** Details the reporting of the reliability analysis

| **Authors and year** | Reliability | Condylar measurements | | | | | | | | Condylar position | | | | |
| --- | --- | --- | --- | --- | --- | --- | --- | --- | --- | --- | --- | --- | --- | --- |
|  |  | Condylar length | Condylar width | Condylar height | Surface area (axial, sagittal or coronal) | Condylar inclination (ML, V or AP) | intercondylar distance (medial, lateral, or geometric) | Condylar volume | General reliability of condylar measurements | Point condyle position (ML, V or AP) | Geometric condyle position (ML, V or AP) | Condyle joint position (ML, V or AP) | General reliability of condylar position | One value or range mentioned for all |
| **García-Díaz et al. 2020^26^** | Inter-examiner reliability | NA | NA | NA | NA | NA | NA | NA | NA | NA | NA | NA | NA | NA |
|  | Intra-examiner reliability | NM | NM | NM | NM | NM | NM | NA | NM | NM | NM | NM | NM | 0.8 |
| **Al-koshab et al. 2015^25^** | Inter-examiner reliability | NA | NA | NA | NA | NA | NA | NA | NA | NA | NA | NA | NA | NA |
|  | Intra-examiner reliability | 0.92 | 0.98 | 0.97 | NM | NM | NM | 0.9 | NM | NM | NM | NM | NM | 0.98 |
| **Huang et al. 2017^29^** | Inter-examiner reliability | NA | NA | NA | NA | NA | NA | NA | NA | NA | NA | NA | NA | NA |
|  | Intra-examiner reliability | NM | NM | NM | NM | NM | NM | NM | NM | NM | NM | NM | NM | 0.971 - 0.993 |
| **Chae et al. 2020^31^** | Inter-examiner reliability | NA | NA | NA | NA | NA | NA | NA | NA | NA | NA | NA | NA | NA |
|  | Intra-examiner reliability | NM | NM | NM | NM | NM | NM | NM | NM | NM | NM | NM | NM | 0.96 – 1 |
| **Lobo et al. 2019^32^** | Inter-examiner reliability | NA | NA | NA | NA | NA | NA | NA | NA | NA | NA | NA | NA | NA |
|  | Intra-examiner reliability | NM | NM | NM | NM | NM | NM | NM | NM | NM | NM | NM | NM | Examiner 1 = 0.999, examiner 2 = 0.995, interexaminer = 0.996 |
| **Alhammadi et al. 2014^27^** | Inter-examiner reliability | 0.951 | 0.931 | 0.945 | 0.925 | 0.89 | 0.91 | NM | NM | 0.92 | 0.896 | 0.899 | NM | 0.79 - 0.993 |
|  | Intra-examiner reliability | 0.993 | 0.953 | 0.975 | 0.955 | 0.903 | 0.93 | NM | NM | 0.933 | 0.926 | 0.903 | NM | 0.79 - 0.993 |
| **Alhammadi et al. 2016a^19^** | Inter-examiner reliability | 0.951 | 0.931 | 0.945 | 0.925 | 0.89 | 0.91 | NM | NM | 0.92 | 0.896 | 0.899 |  | 0.76 - 0.99 |
|  | Intra-examiner reliability | 0.993 | 0.958 | 0.975 | 0.955 | 0.903 | 0.93 | NM | NM | 0.933 | 0.926 | 0.903 | NM | 0.79 - 0.993 |
| **Alhammadi et al. 2016b^20^** | Inter-examiner reliability | NM | NM | NM | NM | NM | NM | NM | NM | NM | NM | NM | NM | 0.79 - 0.993 |
|  | Intra-examiner reliability | NM | NM | NM | NM | NM | NM | NM | NM | NM | NM | NM | NM | 0.79 - 0.993 |
| **Serindere et al. 2020^21^** | Inter-examiner reliability | NM | NM | NM | NM | NM | NM | NM | NM | NM | NM | NM | NM | 0.995 - 0.996 |
|  | Intra-examiner reliability | NM | NM | NM | NM | NM | NM | NM | NM | NM | NM | NM | NM | 0.995 - 0.996 |
| **Endo et al. 2011^22^** | Inter-examiner reliability | NA | NA | NA | NA | NA | NA | NA | NA | NA | NA | NA | NA | NA |
|  | Intra-examiner reliability | NM | NM | NM | NM | NM | NM | NM | NM | NM | NM | NM | NM | 0.9 |
| **Ahmed et al. 2021^24^** | Inter-examiner reliability | NA | NA | NA | NA | NA | NA | NA | NA | NA | NA | NA | NA | NA |
|  | Intra-examiner reliability | NM | NM | NM | NM | NM | NM | NM | NM | NM | NM | NM | NM | 0.9 |
| **Al-hadad et al. 2022^28^** | Inter-examiner reliability | NM | NM | NM | NM | NM | NM | NM | NM | NM | NM | NM | NM | NM |
|  | Intra-examiner reliability | NM | NM | NM | NM | NM | NM | NM | NM | NM | NM | NM | NM | NM |
| **Chen et al. 2022a^23^** | Inter-examiner reliability | NM | NM | NM | NM | NM | NM | NM | NM | NM | NM | NM | NM | 0.97 – 1 |
|  | Intra-examiner reliability | NM | NM | NM | NM | NM | NM | NM | NM | NM | NM | NM | NM | 0.97 – 1 |
| **Chen et al. 2022b^30^** | Inter-examiner reliability | NM | NM | NM | NM | NM | NM | NM | NM | NM | NM | NM | NM | 0.96 – 1 |
|  | Intra-examiner reliability | NM | NM | NM | NM | NM | NM | NM | NM | NM | NM | NM | NM | 0.96 – 1 |

| **Authors and year** | Reliability | Fossa/Tubercle measurements | | | | | | | | | |
| --- | --- | --- | --- | --- | --- | --- | --- | --- | --- | --- | --- |
|  |  | Interfossa distance | Fossa inclination (AP, V or ML) | Fossa position (AP, V or ML) | Surface area (axial, sagittal or coronal) | Fossa height | Fossa width | Anterior tubercle inclination | Posterior tubercle inclination | Tubercle height | General reliability of fossa/tubercle measurements |
| **García-Díaz et al. 2020^26^** | Inter-examiner reliability | NA | NA | NA | NA | NA | NA | NA | NA | NA | NA |
|  | Intra-examiner reliability | NM | NM | NM | NM | NM | NM | NM | NM | NM | NM |
| **Al-koshab et al. 2015^25^** | Inter-examiner reliability | NA | NA | NA | NA | NA | NA | NA | NA | NA | NA |
|  | Intra-examiner reliability | NM | NM | NM | NM | NM | Nm | NM | NM | NM | NM |
| **Huang et al. 2017^29^** | Inter-examiner reliability | NA | NA | NA | NA | NA | NA | NA | NA | NA | NA |
|  | Intra-examiner reliability | NM | NM | NM | NM | NM | NM | NM | NM | NM | NM |
| **Chae et al. 2020^31^** | Inter-examiner reliability | NA | NA | NA | NA | NA | NA | NA | NA | NA | NA |
|  | Intra-examiner reliability | NM | NM | NM | NM | NM | NM | NM | NM | NM | NM |
| **Lobo et al. 2019^32^** | Inter-examiner reliability | NA | NA | NA | NA | NA | NA | NA | NA | NA | NA |
|  | Intra-examiner reliability | NM | NM | NM | NM | NM | NM | NM | NM | NM | NM |
| **Alhammadi et al. 2014^27^** | Inter-examiner reliability | 0.892 | 0.822 | 0.81 | 0.803 | 0.896 | 0.819 | 0.86 | 0.828 | 0.919 | NM |
|  | Intra-examiner reliability | 0.9 | 0.855 | 0.882 | 0.8 | 0.846 | 0.852 | 0.896 | 0.866 | 0.945 | NM |
| **Alhammadi et al. 2016a^19^** | Inter-examiner reliability | NM | NM | NM | NM | NM | NM | NM | NM | 0.945 | NM |
|  | Intra-examiner reliability | NM | NM | NM | NM | NM | NM | NM | NM | 0,919 | NM |
| **Alhammadi et al. 2016b^20^** | Inter-examiner reliability | NM | NM | NM | NM | NM | NM | NM | NM | NM | NM |
|  | Intra-examiner reliability | NM | NM | NM | NM | NM | NM | NM | NM | NM | NM |
| **Serindere et al. 2020^21^** | Inter-examiner reliability | NM | NM | NM | NM | NM | NM | NM | NM | NM | NM |
|  | Intra-examiner reliability | NM | NM | NM | NM | NM | NM | NM | NM | NM | NM |
| **Endo et al. 2011^22^** | Inter-examiner reliability | NA | NA | NA | NA | NA | NA | NA | NA | NA | NA |
|  | Intra-examiner reliability | NM | NM | NM | NM | NM | NM | NM | NM | NM | NM |
| **Ahmed et al. 2021^24^** | Inter-examiner reliability | NA | NA | NA | NA | NA | NA | NA | NA | NA | NA |
|  | Intra-examiner reliability | NM | NM | NM | NM | NM | NM | NM | NM | NM | NM |
| **Al-hadad et al. 2022^28^** | Inter-examiner reliability | NM | NM | NM | NM | NM | NM | NM | NM | NM | NM |
|  | Intra-examiner reliability | NM | NM | NM | NM | NM | NM | NM | NM | NM | NM |
| **Chen et al. 2022a^23^** | Inter-examiner reliability | NM | NM | NM | NM | NM | NM | NM | NM | NM | NM |
|  | Intra-examiner reliability | NM | NM | NM | NM | NM | NM | NM | NM | NM | NM |
| **Chen et al. 2022b^30^** | Inter-examiner reliability | NM | NM | NM | NM | NM | NM | NM | NM | NM | NM |
|  | Intra-examiner reliability | NM | NM | NM | NM | NM | NM | NM | NM | NM | NM |

| **Authors and year** | Reliability | Joint space measurements | | | | |  |  |
| --- | --- | --- | --- | --- | --- | --- | --- | --- |
|  |  | Anterior joint space | Posterior joint space | Superior joint space | Medial joint space | General reliability of condylar position | One value or range mentioned for all | Note |
| **García-Díaz et al. 2020^26^** | Inter-examiner reliability | NA | NA | NA | NA | NA | NA |  |
|  | Intra-examiner reliability | NM | NM | NM | NM | NM | 0.8 |  |
| **Al-koshab et al. 2015^25^** | Inter-examiner reliability | NA | NA | NA | NA | NA | NA |  |
|  | Intra-examiner reliability | 0.93 | 0.99 | 0.99 | NM | NM | 0.98 |  |
| **Huang et al. 2017^29^** | Inter-examiner reliability | NA | NA | NA | NA | NA | NA |  |
|  | Intra-examiner reliability | NM | NM | NM | NM | NM | 0.971 - 0.993 |  |
| **Chae et al. 2020^31^** | Inter-examiner reliability | NA | NA | NA | NA | NA | NA |  |
|  | Intra-examiner reliability | NM | NM | NM | NM | NM | 0.96 - 1 |  |
| **Lobo et al. 2019^32^** | Inter-examiner reliability | NA | NA | NA | NA | NA | NA |  |
|  | Intra-examiner reliability | NM | NM | NM | NM | NM | Examiner 1 = 0.999, examiner 2 = 0.995, interexaminer = 0.996 |  |
| **Alhammadi et al. 2014^27^** | Inter-examiner reliability | 0.928 | 0.935 | 0.903 | 0.906 | NM | 0.76 - 0.993 |  |
|  | Intra-examiner reliability | 0.958 | 0.955 | 0.925 | 0.934 | NM | 0.79 - 0.993 |  |
| **Alhammadi et al. 2016a^19^** | Inter-examiner reliability | 0.928 | 0.935 | 0.903 | 0.906 | NM | 0.76 - 0.99 |  |
|  | Intra-examiner reliability | 0.958 | 0.955 | 0.925 | 0.934 |  | 0.79 - 0.993 |  |
| **Alhammadi et al. 2016b^20^** | Inter-examiner reliability | NM | NM | NM | NM | NM | 0.79 - 0.93 |  |
|  | Intra-examiner reliability | NM | NM | NM | NM | NM | 0.79 - 0.93 |  |
| **Serindere et al. 2020^21^** | Inter-examiner reliability | NM | NM | NM | NM | NM | 0.995 - 0.996 |  |
|  | Intra-examiner reliability | NM | NM | NM | NM | NM | 0.995 - 0.996 |  |
| **Endo et al. 2011^22^** | Inter-examiner reliability | NA | NA | NA | NA | NA | NA |  |
|  | Intra-examiner reliability | NM | NM | NM | NM | NM | 0.9 |  |
| **Ahmed et al. 2021^24^** | Inter-examiner reliability | NA | NA | NA | NA | NA | NA |  |
|  | Intra-examiner reliability | NM | NM | NM | NM | NM | 0.9 |  |
| **Al-hadad et al. 2022^28^** | Inter-examiner reliability | NM | NM | NM | NM | NM | NM | Lanmarks rather than measurements' reliabilty |
|  | Intra-examiner reliability | NM | NM | NM | NM | NM | NM |  |
| **Chen et al. 2022a^23^** | Inter-examiner reliability | NM | NM | NM | NM | NM | 0.97 - 1 |  |
|  | Intra-examiner reliability | NM | NM | NM | NM | NM | 0.97 - 1 |  |
| **Chen et al. 2022b^30^** | Inter-examiner reliability | NM | NM | NM | NM | NM | 0.96 - 1 |  |
|  | Intra-examiner reliability | NM | NM | NM | NM | NM | 0.96 - 1 |  |
|  |  |  |  |  |  |  |  |  |
| NA = Not Applicable |  |  |  |  |  |  |  |  |
| NM = Not Mentioned |  |  |  |  |  |  |  |  |

**Supplementary VI:** PRISMA checklist.

| **Section and Topic** | **Item #** | **Checklist item** | **Location where item is reported** |
| --- | --- | --- | --- |
| **TITLE** | | |  |
| Title | 1 | Identify the report as a systematic review. | 1 |
| **ABSTRACT** | | |  |
| Abstract | 2 | See the PRISMA 2020 for Abstracts checklist. | 1 |
| **INTRODUCTION** | | |  |
| Rationale | 3 | Describe the rationale for the review in the context of existing knowledge. | 2 |
| Objectives | 4 | Provide an explicit statement of the objective(s) or question(s) the review addresses. | 3 |
| **METHODS** | | |  |
| Eligibility criteria | 5 | Specify the inclusion and exclusion criteria for the review and how studies were grouped for the syntheses. | 3 |
| Information sources | 6 | Specify all databases, registers, websites, organisations, reference lists and other sources searched or consulted to identify studies. Specify the date when each source was last searched or consulted. | 4 |
| Search strategy | 7 | Present the full search strategies for all databases, registers and websites, including any filters and limits used. | 4 |
| Selection process | 8 | Specify the methods used to decide whether a study met the inclusion criteria of the review, including how many reviewers screened each record and each report retrieved, whether they worked independently, and if applicable, details of automation tools used in the process. | 4 |
| Data collection process | 9 | Specify the methods used to collect data from reports, including how many reviewers collected data from each report, whether they worked independently, any processes for obtaining or confirming data from study investigators, and if applicable, details of automation tools used in the process. | 4 |
| Data items | 10a | List and define all outcomes for which data were sought. Specify whether all results that were compatible with each outcome domain in each study were sought (e.g. for all measures, time points, analyses), and if not, the methods used to decide which results to collect. | 5 |
|  | 10b | List and define all other variables for which data were sought (e.g. participant and intervention characteristics, funding sources). Describe any assumptions made about any missing or unclear information. | 5 |
| Study risk of bias assessment | 11 | Specify the methods used to assess risk of bias in the included studies, including details of the tool(s) used, how many reviewers assessed each study and whether they worked independently, and if applicable, details of automation tools used in the process. | 5 |
| Effect measures | 12 | Specify for each outcome the effect measure(s) (e.g. risk ratio, mean difference) used in the synthesis or presentation of results. | 5 |
| Synthesis methods | 13a | Describe the processes used to decide which studies were eligible for each synthesis (e.g. tabulating the study intervention characteristics and comparing against the planned groups for each synthesis (item #5)). | 5 |
|  | 13b | Describe any methods required to prepare the data for presentation or synthesis, such as handling of missing summary statistics, or data conversions. | NA |
|  | 13c | Describe any methods used to tabulate or visually display results of individual studies and syntheses. | NA |
|  | 13d | Describe any methods used to synthesize results and provide a rationale for the choice(s). If meta-analysis was performed, describe the model(s), method(s) to identify the presence and extent of statistical heterogeneity, and software package(s) used. | NA |
|  | 13e | Describe any methods used to explore possible causes of heterogeneity among study results (e.g. subgroup analysis, meta-regression). | NA |
|  | 13f | Describe any sensitivity analyses conducted to assess robustness of the synthesized results. | NA |
| Reporting bias assessment | 14 | Describe any methods used to assess risk of bias due to missing results in a synthesis (arising from reporting biases). | 5 |
| Certainty assessment | 15 | Describe any methods used to assess certainty (or confidence) in the body of evidence for an outcome. | NA |
| **RESULTS** | | |  |
| Study selection | 16a | Describe the results of the search and selection process, from the number of records identified in the search to the number of studies included in the review, ideally using a flow diagram. | 6 |
|  | 16b | Cite studies that might appear to meet the inclusion criteria, but which were excluded, and explain why they were excluded. | 6 |
| Study characteristics | 17 | Cite each included study and present its characteristics. | 6 |
| Risk of bias in studies | 18 | Present assessments of risk of bias for each included study. | 7 |
| Results of individual studies | 19 | For all outcomes, present, for each study: (a) summary statistics for each group (where appropriate) and (b) an effect estimate and its precision (e.g. confidence/credible interval), ideally using structured tables or plots. | 7 |
| Results of syntheses | 20a | For each synthesis, briefly summarise the characteristics and risk of bias among contributing studies. | NA |
|  | 20b | Present results of all statistical syntheses conducted. If meta-analysis was done, present for each the summary estimate and its precision (e.g. confidence/credible interval) and measures of statistical heterogeneity. If comparing groups, describe the direction of the effect. | NA |
|  | 20c | Present results of all investigations of possible causes of heterogeneity among study results. | NA |
|  | 20d | Present results of all sensitivity analyses conducted to assess the robustness of the synthesized results. | NA |
| Reporting biases | 21 | Present assessments of risk of bias due to missing results (arising from reporting biases) for each synthesis assessed. | NA |
| Certainty of evidence | 22 | Present assessments of certainty (or confidence) in the body of evidence for each outcome assessed. | NA |
| **DISCUSSION** | | |  |
| Discussion | 23a | Provide a general interpretation of the results in the context of other evidence. | 9 |
|  | 23b | Discuss any limitations of the evidence included in the review. | 9,10 |
|  | 23c | Discuss any limitations of the review processes used. | 11 |
|  | 23d | Discuss implications of the results for practice, policy, and future research. | 11 |
| **OTHER INFORMATION** | | |  |
| Registration and protocol | 24a | Provide registration information for the review, including register name and registration number, or state that the review was not registered. | 1 |
|  | 24b | Indicate where the review protocol can be accessed, or state that a protocol was not prepared. |  |
|  | 24c | Describe and explain any amendments to information provided at registration or in the protocol. |  |
| Support | 25 | Describe sources of financial or non-financial support for the review, and the role of the funders or sponsors in the review. | 12 |
| Competing interests | 26 | Declare any competing interests of review authors. | 12 |
| Availability of data, code and other materials | 27 | Report which of the following are publicly available and where they can be found: template data collection forms; data extracted from included studies; data used for all analyses; analytic code; any other materials used in the review. | 12 |

*From:*  Page MJ, McKenzie JE, Bossuyt PM, Boutron I, Hoffmann TC, Mulrow CD, et al. The PRISMA 2020 statement: an updated guideline for reporting systematic reviews. BMJ 2021;372:n71. doi: 10.1136/bmj.n71

For more information, visit: <http://www.prisma-statement.org/>
